# 

**Published:** 1864-01

**Authors:** 


*Contents.*

ORIGINAL COMMUNICATIONS........................................ 1

1. Traumatic Tetanus, by Prof. Jos Jones. 2. Resections of the Hip-Joint, by Surg James B. Read. 3. On the External Application of Oil of Turpentine as a Substitute for Quinine in Intermittent Fever, with Reports of Cases. 4 Case of Tetanus—Recovery—reported by Surgeon W. A. Davis.

CONFEDERATE STATES HOSPITAL REPORTS............... 8

1. Statistics of Winder Hospital, Richmond, Virginia, from its organization, April 1, 1862, to December 1, 1863. 2. Statistics of Chimborazo Hospital, from November 1, 1861, to November 1, 1863. 3. Statistics of Compound Fracture of Femur—A report of cases of Compound Comminuted Fracture of the Thigh from Gun-Shot Wounds treated in Winder Hospital, Richmond, Va., since its organization, April 1, 1862, to Dec'r 1, 1863. 4. Report of cases of Gun Shot Fracture of Femur treated without operative procedure, Chimborazo Hospital.

CHRONICLE OF MEDICAL SCIENCE............................. 11

Mortality Rates of the U. S. Armies for the Year ending June, '62, by Surgeon J. J. Woodward, U. S. A.

EDITORIAL AND MISCELLANEOUS................................ 13

Salutatory. Association of Army and Navy Surgeons. To Contributors.

